# Perovskites to Photonics: Engineering NIR LEDs for Photobiomodulation

**DOI:** 10.3390/mi16091002

**Published:** 2025-08-30

**Authors:** Somnath Mahato, Hendradi Hardhienata, Muhammad Danang Birowosuto

**Affiliations:** 1 Łukasiewicz Research Network-PORT Polish Center for Technology Development, Stabłowicka 147, 54-066 Wrocław, Poland; somnath.mahato@port.lukasiewicz.gov.pl; 2 Theoretical Physics Division, Department of Physics, IPB University, Meranti Avenue, Wing S Building, Dramaga Campus of IPB, Bogor 16680, West Java, Indonesia; hendradi@apps.ipb.ac.id

**Keywords:** near infra red, LED, photobiomodulation, perovskites, photonics

## Abstract

Photobiomodulation (PBM) harnesses near-infrared (NIR) light to stimulate cellular processes, offering non-invasive treatment options for a range of conditions, including chronic wounds, inflammation, and neurological disorders. NIR light-emitting diodes (LEDs) are emerging as safer and more scalable alternatives to conventional lasers, but optimizing their performance for clinical use remains a challenge. This perspective explores the latest advances in NIR-emitting materials, spanning Group III–V, IV, and II–VI semiconductors, organic small molecules, polymers, and perovskites, with an emphasis on their applicability to PBM. Particular attention is given to the promise of perovskite LEDs, including lead-free and lanthanide-doped variants, for delivering narrowband, tunable NIR emission. Furthermore, we examine photonic and plasmonic engineering strategies that enhance light extraction, spectral precision, and device efficiency. By integrating advances in materials science and nanophotonics, it is increasingly feasible to develop flexible, biocompatible, and high-performance NIR LEDs tailored for next-generation therapeutic applications.

## 1. Introduction

Photobiomodulation (PBM) is a non-invasive, non-thermal therapeutic approach that employs non-ionizing light to stimulate beneficial biological processes at the cellular and tissue levels. Near-infrared (NIR) light-emitting diodes (LEDs) have emerged as particularly effective tools for PBM, because they combine deep tissue penetration with precise cellular-level interaction and minimal side effects. PBM operates through photon absorption by chromophores such as cytochrome c oxidase (COX), which enhances cellular metabolism, reduces inflammation, and promotes tissue regeneration [1,2,3]. This therapeutic approach typically employs LEDs and lasers that emit light across the visible (400–700 nm) and NIR (700–1350 nm) spectra [4,5,6], thereby linking fundamental photobiological mechanisms to practical clinical outcomes. NIR wavelengths, especially NIR I (700–1000 nm), are the most effective one for clinical applications such as chronic wound healing, transcranial neurostimulation, and retinal disease healing [4,7,8].

In addition to therapeutic benefits, NIR light allows precise monitoring and modulation of cellular activity with subcellular spatial resolution and sub-millisecond temporal accuracy [9]. Its ability to penetrate deeply into tissues with minimal scattering and photodamage enables researchers to study dynamic cellular processes, manipulate neural or biochemical pathways, and achieve targeted therapeutic interventions. These characteristics translate directly into clinical advantages, including dermatology, neurology, and ophthalmology. Collectively, these properties highlight the unique potential of NIR LEDs to bridge fundamental photobiological effects with clinical applications.

Realizing this potential requires the development of novel NIR-emitting materials that are efficient, biocompatible, and environmentally friendly. Advanced NIR light sources optimized for interaction with biological systems can expand PBM to new clinical and biomedical applications. By integrating innovations in materials, precise light delivery, and mechanistic understanding, NIR light-based approaches create a seamless pathway from fundamental research to transformative precision medicine.

Traditional inorganic LEDs, based on Group III–V, IV, and II–VI semiconductors, provide high radiative efficiency but are limited by high production costs, rigidity, and toxicity [6]. Emerging technologies that utilize small molecules and conjugated polymers offer flexible and low-cost alternatives, although stability and efficiency in the NIR region remain challenging [10]. Perovskite LEDs, particularly those that use lead halide nanocrystals, exhibit excellent performance in the visible spectrum due to their high photoluminescence quantum yield (PLQY) and tunable emission [11,12]. However, extending these advantages to NIR emission is still challenging, which has driven the development of lead-free alternatives such as tin-based perovskites, double perovskites, and lanthanide-doped halides [11,12,13].

**Figure 1:**
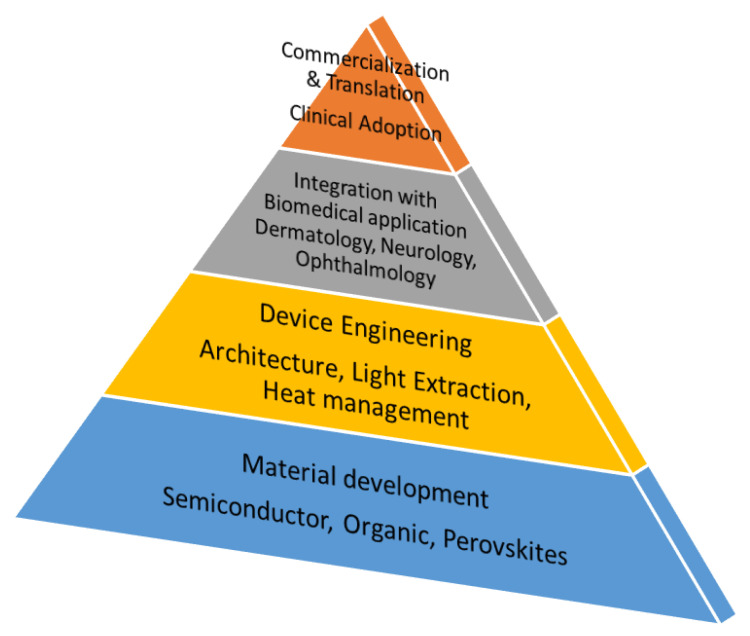
Roadmap for photobiomodulation (PBM) based on the material development and device engineering.

Beyond material innovations, photonic and plasmonic engineering can further optimize NIR LED performance. Nanostructured electrodes, optical cavities, and surface plasmon coupling improve light outcoupling, spectral precision, and thermal management [14,15]. Taken together, advances in both materials and photonic design provide a comprehensive strategy to enhance NIR LEDs for PBM, as seen in the roadmap of Figure 1. The convergence of optoelectronics and biomedicine in this perspective represents a transformative opportunity for next-generation therapeutic technologies.

## 2. NIR Emission for Photobiomodulation

### 2.1. Biological Basis of PBM

Understanding the biological basis of PBM is essential before discussing device design. PBM employs red (630–700 nm), NIR-I (700–1000 nm), and NIR-II (1000–1350 nm) to modulate cellular activity without thermal damage. However, in our discussion, we would like to limit our discussion to NIR-I as it is the region with the most benefits for clinical and preclinical studies, including wound healing, reduced inflammation, pain relief, brain therapy, and retinal diseases [16]. The primary mechanism involves the absorption of photons by COX, leading to photodissociation of nitric oxide, enhanced electron transport, and increased ATP production [2,16]. These events promote cell proliferation, regulate cytokine expression, and improve perfusion. The “optical window” (700–950 nm) is especially effective for tissue penetration, with 800–950 nm wavelengths most suitable for deeper targets [2,17].

### 2.2. Clinical Applications

**Figure 2:**
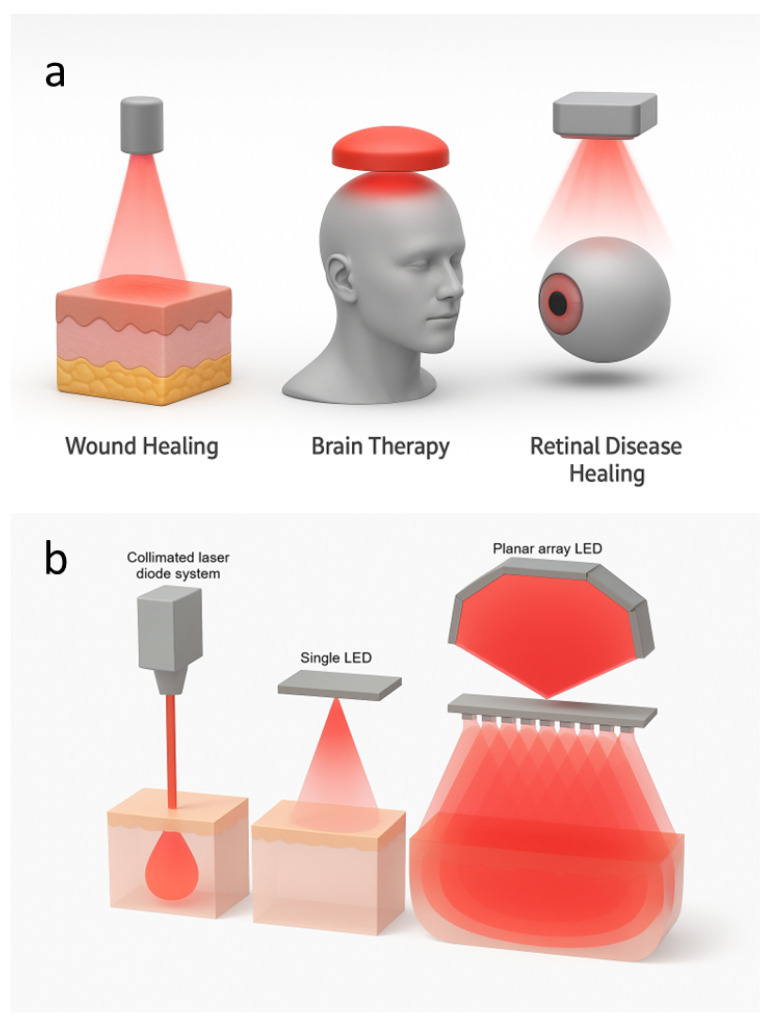
Near-infrared (NIR) PBM using light-based systems. (**a**) Applications of light therapy, including wound healing, brain therapy, and retinal disease healing. (**b**) Comparison of light delivery methods: a collimated laser diode, a single light-emitting diode (LED), and a planar array LED.

Clinical evidence highlights PBM’s broad therapeutic potential across dermatology, neurology, and ophthalmology, see Figure 2a. In the brain, transcranial PBM has shown promise in Alzheimer’s disease, traumatic brain injury, and ischemic stroke, where mechanisms include vasodilation, enhanced neurogenesis, mitochondrial support, and antiapoptotic signaling that contribute to better neural recovery [8]. In dermatology and wound healing, PBM accelerates tissue repair by stimulating re-epithelialization, suppressing pro-inflammatory cytokines such as IL-1 and TNF-α, and promoting angiogenesis and extracellular matrix remodeling, leading to faster closure of chronic ulcers and improved scar quality [16,17]. In ophthalmology, PBM represents a non-invasive strategy for retinal diseases, with clinical studies reporting gains in visual acuity and reduced pathological changes in age-related macular degeneration and diabetic macular edema [18,19,20]. Preclinical and early clinical evidence also supports the benefits in retinopathy of prematurity, amblyopia beyond the critical period, retinitis pigmentosa, and methanol-induced retinal toxicity [21,22,23,24]. Taken together, these findings position PBM as a versatile and cost-effective adjunctive therapy with translational relevance in the major domains of medicine, while underscoring the need for standardized protocols and large-scale trials to validate long-term efficacy.

### 2.3. Light Sources: Lasers vs. LEDs

PBM primarily employs lasers and LEDs as light sources. As illustrated in Figure 2b, lasers generate a focused coherent beam that enables deep tissue penetration and precise fluence delivery, whereas LEDs emit broader and less intense light. A single LED provides wide but shallow illumination, while an LED array produces overlapping beams that enhance scattering and create larger regions of high photon density. These different LED configurations are used in PBM to target different therapeutic applications. Although lasers offer superior coherence and penetration, LEDs are generally preferred for their safety, affordability, and suitability for large-area or wearable treatments. LEDs also minimize thermal and ocular risks, making them particularly advantageous for long-term or self-administered use. Recent developments in flexible, skin-conformal LED arrays further support efficient, low-cost, and personalized PBM therapies [7].

### 2.4. Device Metrics and Dosimetry

Reproducible PBM requires linking biological outcomes with measurable device parameters. Key metrics include irradiance ($\mathrm{mW}/{\mathrm{cm}}^{2}$), the optical power per unit area [25]; radiant exposure ($J/{\mathrm{cm}}^{2}$), the cumulative dose defined as H=E·t [26]; and duty cycle (%), the fraction of time a source emits within each pulse cycle [25]. These quantities provide a common framework for translating clinical prescriptions into engineering design choices.

### 2.5. Translating Clinical Protocols into Device Parameters

Clinical PBM protocols are typically reported using wavelength, fluence, irradiance, treatment time, and duty cycle. In contrast, device engineers manipulate emitter density, panel area, diode drive current, and pulsing schemes. Establishing a mapping between these domains is critical for safety and reproducibility. For example, a clinical protocol specifying 810 nm at 50 mW/cm^2^ for 2 min of continuous-wave (CW) illumination can be implemented by tuning diode current, emitter spacing, and optical layout. If pulsed-wave (PW) operation is used (e.g., 50% duty cycle), either the session time or instantaneous irradiance must be doubled to deliver the same radiant exposure. Because PBM outcomes follow a biphasic dose–response, both under-dosing and over-dosing risk reducing therapeutic benefit [27].

Table 1 illustrates the diversity of PBM protocols across medical domains. In neurology, dosing spans from low-fluence CW delivery in acute stroke (808 nm, 1.2 J/cm^2^) [28] to higher fluences in TBI (810–980 nm, 14.8–28.3 J/cm^2^ with PW at 10 Hz) [29], and very high doses in depressive disorders (810 nm, 60 J/cm^2^) [30]. In wound care, PBM protocols vary by depth and severity: superficial burns use 3 J/cm^2^ at 785–830 nm [31], dermal wounds employ 1–6 J/cm^2^ at 810–830 nm [2], and deep diabetic wounds require pulsed 904 nm light at high irradiance (up to 18.3 J/cm^2^) [5,32]. Retinal applications typically employ near-infrared light between 780 and 830 nm to avoid photothermal damage. Clinical studies demonstrate improvements in visual acuity and scotoma reduction in age-related macular degeneration [18], while additional reports show benefits in amblyopia and retinitis pigmentosa [22,23]. Together, these data emphasize how wavelength, fluence, and duty cycle must be carefully tuned not only to tissue depth and pathology but also to the optical safety constraints of the retina.

### 2.6. Thermal Load and Worked Example

Thermal management is a central engineering challenge. Heating depends on irradiance, wavelength, beam profile, and duty cycle. For example, 980 nm sources typically generate more heat than 810 nm sources, and Gaussian beams concentrate hotspots compared to flat-top beams [33]. Furthermore, as seen on Table 2, we consider a diode with radiance $L=10\;W\;{\mathrm{sr}}^{-1}\;{\mathrm{cm}}^{-2}$ and Lambertian emission (half-angle 60°) illuminating 1 cm^2^. The irradiance is $E=L\cdot \pi \approx 31\;W/{\mathrm{cm}}^{2}$ (31,000 mW/cm^2^). With a 50% duty cycle, the average irradiance is 16,000 mW/cm^2^. Over 60 s, this corresponds to 940 J/cm^2^, far above therapeutic ranges. Such calculations highlight the need to balance duty cycle, beam divergence, and exposure duration to ensure safety.

### 2.7. Challenges and Limitations

**Figure 3:**
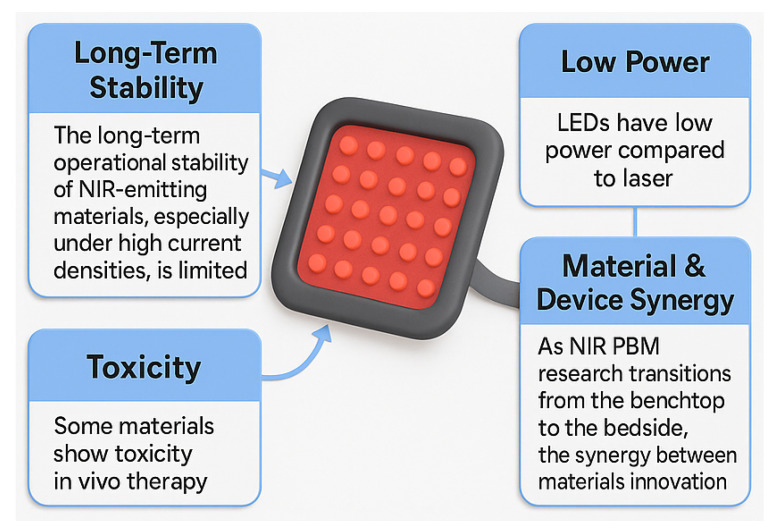
Obstacles in NIR LED for PBM.

Despite progress, obstacles remain. Figure 3 illustrates device-level challenges such as the limited operational stability of NIR-emitting materials, particularly perovskite LEDs at high current densities. Accurate control of wavelength, irradiance, and dosage is also critical; deviations from prescribed values can compromise efficacy or cause adverse effects [2]. Superpulsed NIR LEDs (e.g., 904 nm nanosecond-scale pulses) represent one strategy to improve penetration while reducing thermal load.

### 2.8. Future Directions

Integrating biological understanding, clinical validation, and quantitative dosimetry will guide the development of standardized PBM devices. Emerging technologies such as perovskite-based NIR LEDs and wearable platforms with biosensor feedback promise personalized closed-loop therapies. Progress will require collaboration between materials science, device engineering, and medicine to fully realize the therapeutic potential of PBM.

## 3. NIR LED Emissive Materials

**Figure 4:**
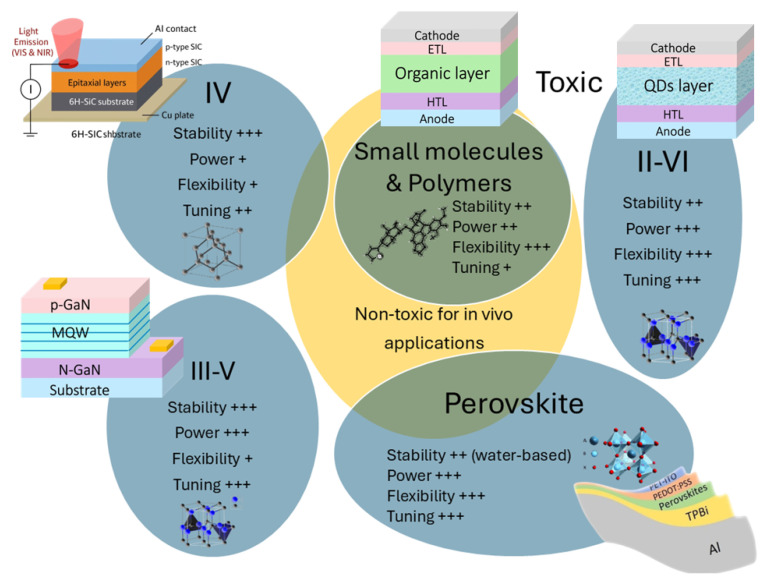
Types of NIR LED emissive materials. Several factors including stability, power, flexibility, tuning, and toxicity as depicted in Figure 3 are considered.

This section consists of four major classes of materials for NIR LEDs in PBM applications: conventional semiconductors, small molecules, polymers, and perovskites. Figure 4 provides an overview of NIR LED materials.

### 3.1. Conventional Semiconductors (Groups III–V, IV, II–VI)

First, group III–V semiconductors such as GaAs, InGaAs, and AlGaAs exhibit direct bandgaps and high radiative efficiencies across 700–950 nm [34,35], resulting in large power. These materials are used in high-power NIR LEDs for clinical applications, including wound healing and neurostimulation [36]. However, epitaxial growth on rigid substrates limits their applicability to flexible and wearable devices [37]. Then, group IV semiconductors (e.g., SiC) have indirect bandgaps, making them inefficient emitters under standard conditions [38]. Nonetheless, strain engineering and nanostructuring have improved SiC-based NIR LEDs, particularly for telecom and bioimaging applications [38,39]. Finally, group II–VI semiconductors, such as CdSe, ZnTe, and CdHgSe, can be tuned for NIR emission via quantum dot engineering and alloying [40,41]. This latest semiconductor offers the best integration with the flexible polymer substrate, but has worse stability compared to the other two. For tuning, similar to III–V semiconductors with pnictogen anions, II–VI materials can be tuned by adjusting the composition of chalcogenide anions, respectively.

### 3.2. Small-Molecule and Polymer LEDs

Organic NIR LEDs based on small molecules and conjugated polymers offer flexibility, low-cost fabrication, and biocompatibility. Small-molecule NIR OLEDs utilize donor–acceptor architectures to achieve emissions beyond 700 nm [11]. Despite progress, nonradiative losses and limited stability remain challenges. Molecular engineering approaches such as backbone rigidification and aggregation suppression have improved an external quantum efficiency (EQE) up to 10% in the 800–850 nm range [42,43,44].

Polymer-based NIR LEDs are well-suited for large-area, skin-conformal devices. Low-bandgap copolymers incorporating benzodithiophene, diketopyrrolopyrrole, or isoindigo units enable emissions between 700 and 950 nm [45]. Techniques like orthogonal solvent processing and host–guest blending improve morphology and device stability [46,47]. Though the current EQE (4%) is modest, they align with the low irradiance levels (100 mW/cm^2^) required for PBM [48]. However, the best EQE of polymer-based NIR LED currently relies on interfacial energy transfer, which is approximately 20% [49].

Both small molecules and polymers are significantly more flexible than group III–V and group IV semiconductors, but they are less stable in comparison. In terms of wavelength tuning, these organic emitters exhibit broadband emission due to their molecular transitions, making them less straightforward than the inorganic semiconductors, as their emission properties depend heavily on the ligands. However, this behavior could change if they are doped with lanthanides.

### 3.3. Perovskite LEDs

**Figure 5:**
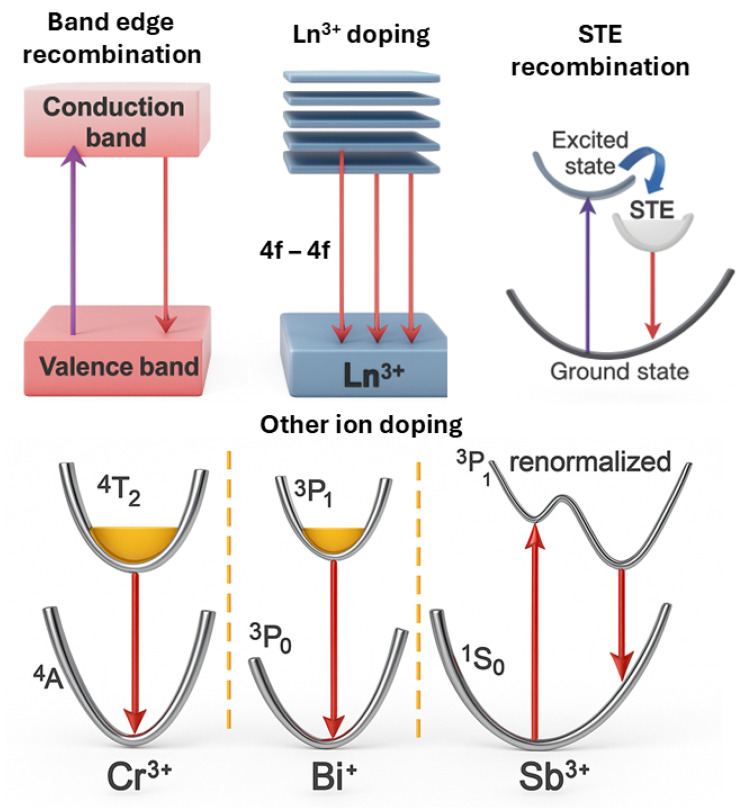
Conceptual drawings of the mechanisms driving NIR emission in perovskite materials. The purple, red, and blue arrows correspond to the absorption, emission, and energy transfer process, respectively.

Perovskite-based materials have emerged as versatile candidates for NIR LEDs owing to their tunable bandgaps, high PLQY, and facile fabrication through solution processing [50,51]. Some progresses in perovskite NIR LEDs were already discussed by Liu et al. [9] while they are depicted in Figure 5. In lead-halide perovskites such as CsPbI_3_ and FAPbI_3_, NIR emission typically arises from band-edge recombination between the conduction band minimum and valence band maximum, yielding narrowband luminescence in the 700–800 nm range [52,53]. Tin-based analogs, such as CsSnI_3_, extend the emission toward 950 nm, though the instability of Sn^2+^ due to oxidation remains a critical issue [54]. Emission wavelength and device efficiency can be further tuned through dimensional engineering, compositional alloying, and surface passivation strategies [9].

Lanthanide ion doping (Ln^3+^) (e.g., Yb^3+^, Nd^3+^, Er^3+^) introduces sharp atomic-like NIR emissions through 4f transitions, typically in the 900–1600 nm range [55]. Quantum cutting mechanisms in Yb^3+^-doped systems have yielded the PLQY that exceed 100% [56]. However, PLQY values above 100% obtained under photoluminescence excitation do not directly imply high EQE under electroluminescence. Efficient EL requires not only radiative efficiency at Yb^3+^ centers but also effective carrier injection and transfer to these sites. Without optimized carrier pathways, the EQE of devices can remain much lower than the PLQY. Co-doping and host lattice optimization, including lead-free alternatives like Cs_2_AgBiBr_6_, have shown promise in mitigating toxicity while preserving NIR performance [57]. However, the intrinsically low absorption cross-sections of 4f transitions necessitate sensitizer ions or energy transfer mechanisms to enable efficient excitation [55].

In addition to narrowband emitters, broadband NIR emission can be achieved through self-trapped exciton (STE) recombination, as observed in vacancy-ordered or low-dimensional perovskites like DFPD_2_CsBiI_6_ and Bmpip_2_SnI_4_ [58,59]. These STEs arise from strong electron–phonon coupling and lattice distortion, offering broad emission suitable for diffuse illumination in PBM or imaging [60]. Optimizing lattice rigidity and minimizing nonradiative losses are key to improving the PLQY in such systems.

Moreover, doping with transition or heavy metal ions, such as Cr^3+^, Bi^+^/Bi^3+^, or Sb^3+^, creates localized states in the bandgap, enabling additional NIR transitions. Cr^3+^-doped double perovskites have achieved emissions from 958 to 1010 nm with a PLQY up to 23% [61,62]. Bi^+^ and Sb^3+^ dopants offer emissions in the 900–1015 nm range with efficiencies as high as 70% [9,63].

Perovskite NIR LEDs based on these mechanisms have reached an EQE exceeding 20% in some band-edge-emission devices [64], and continued progress in co-doping and hybrid emission strategies could further expand the spectral range and application space [58,59,65]. Perovskite materials exhibit similar NIR LED properties to those of small molecules and polymers, see Figure 4. However, like III–V and II–VI semiconductors, their emission wavelength can be easily tuned by adjusting the composition of halide ions. Although the emission remains broad, incorporating lanthanide dopants may help in achieving more precise wavelength tuning.

Despite rapid progress, perovskite NIR LEDs still face key bottlenecks, including nonradiative recombination at defect sites, ion migration, and limited operational lifetimes relative to III–V devices [54,64]. Unencapsulated devices often show T_5_ values of only a few to tens of hours, though improved interfaces and compositions have extended lifetimes of 100 to 300 h [12,54]. For clinical applications of PBM, stability and encapsulation are critical, with >1000 h under 85 °C/85% RH recognized as a benchmark [66,67]. Hybrid polymer–inorganic encapsulation shows promise, but scalable solutions are needed [66]. Photonic strategies such as photonic crystals or microlens arrays can increase outcoupling by 1.5–2×, highlighting their essential role [68,69].

### 3.4. Non-Toxic Emitters

Toxicity remains a major consideration for in vivo applications, see Figure 4. Most III–V and II–VI semiconductor materials [70] are toxic due to the presence of As, Cd, or Hg, while group IV materials such as SiC are generally considered safe, though some derivatives or fabrication processes may pose risks [39]. In contrast, small-molecule and polymer LEDs are largely composed of carbon-based compounds and are thus more biocompatible [66]. However, their limited optical power output can restrict their effectiveness in PBM applications.

### 3.5. Comparative Performance Analysis

For PBM applications, device metrics must extend beyond those typically considered for display and lighting. Critical parameters include the emission peak ($\lambda_{\mathrm{peak}}$) within the optical therapeutic window, spectral full-width at half maximum (FWHM), external quantum efficiency (EQE), wall-plug efficiency (WPE), and the radiance or irradiance at the treatment plane. Reliability under operation is assessed by the current density at EQE roll-off ($J_{\mathrm{ro}}$), spectral drift during continuous drive (S), and the device half-lifetime ($LT_{50}$) under clinically relevant test conditions. Additionally, for wearable PBM systems, the surface temperature rise (Δ*T*) during operation is a key safety and comfort parameter.

Table 3 summarizes the reported performance of representative LED device classes suitable for NIR-PBM. The values consolidate data from recent literature on PBM light sources [10,11,12,67,71,72] and emerging optoelectronic materials, together with device physics evaluations from display and lighting research.

Although III–V semiconductors remain the benchmark for efficiency and operational stability, their rigid and expensive fabrication limits widespread deployment in wearable devices. Group IV silicon-based emitters offer CMOS compatibility yet are fundamentally limited in radiative efficiency. Groups II–VI quantum dot LEDs and perovskite LEDs deliver attractive spectral control and solution-processability, though both face challenges in stability. Organic small-molecule and polymer LEDs enable mechanical flexibility but generally suffer from low efficiency and short operational lifetimes. Looking forward, the most promising strategies may involve hybrid integration, combining stable inorganic emitters (III–V or II–VI) with lightweight flexible substrates, or advancing perovskite and QLED technologies for cost-effective, spectrally precise, and large-area PBM systems.

From all materials, we believe that perovskites offer a promising path forward, combining the solution processability and the long spectral tunability while lead-free-based perovskites have improved safety profiles. Systems such as Cs_2_AgBiBr_6_ and Cs_2_NaInCl_6_ doped with lanthanide or transition metal ions have shown the potential for NIR emission without toxicity concerns [9]. Nonetheless, the field of lead-free perovskite NIR LEDs remains in its infancy, with considerable room for performance optimization. Ongoing material development, including improvements in phase stability, PLQY, and energy transfer efficiency, is expected to unlock their full potential for safe and effective PBM.

## 4. Photonic, Plasmonic and Optoelectronic Engineering for NIR LEDs

**Figure 6:**
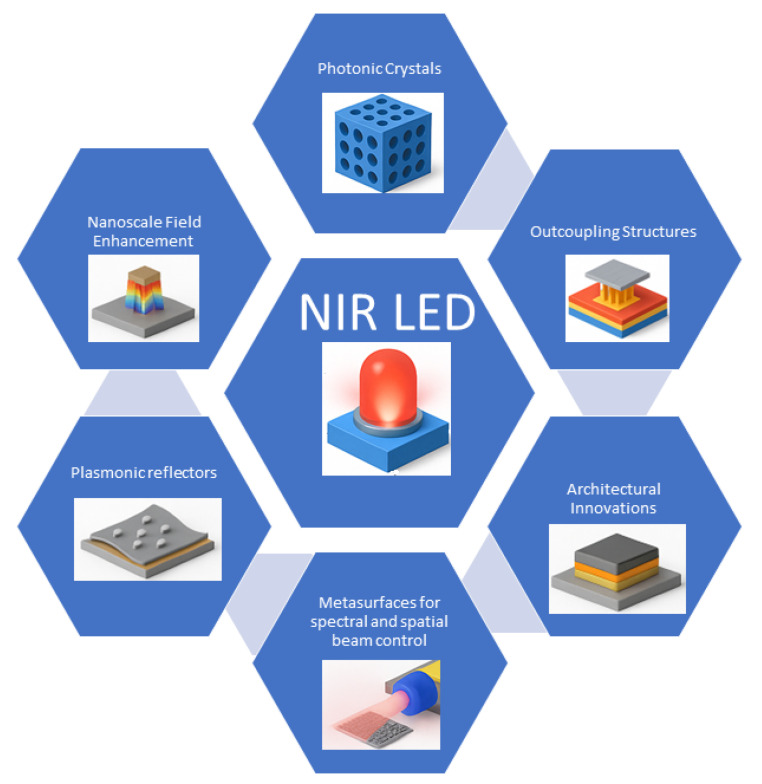
Key components for photonic, plasmonic, and optoelectronic engineering of NIR LEDs.

Photonic, plasmonic, and optoelectronic engineering strategies are vital to enhancing the performance of NIR LEDs, particularly for PBM applications, as seen in Figure 6. These approaches aim to overcome limitations such as nonradiative recombination, emitter aggregation, and energy-gap law constraints [73]. Nanostructures like photonic crystals and plasmonic arrays can localize electromagnetic fields at the nanoscale, enhancing spontaneous emission via near-field interactions [74,75,76,77,78]. Complementary strategies, including dipole orientation alignment [79,80] and light outcoupling designs [68], further mitigate optical losses and improve the external EQE.

Photonic crystals, developed as waveguides, light extraction surfaces, cavities for lasers, and biosensing elements, have been adapted for NIR LED platforms to achieve optical feedback and spectral refinement [81]. Similarly, III–V based light sources integrated with photonic crystal cavities present opportunities for narrowband and directional PBM sources [82,83]. On the emitter front, ZnF_2_ shell passivation of II–VI and III–V quantum dots significantly enhances the PLQY by reducing surface trap states [84]. Coupling this with in situ photo-crosslinked transport layers leads to better charge balance and device stability, with the reported EQE exceeding 20% [84].

Plasmonic enhancements using embedded nanostructures, such as silver nanoparticles in flexible polymers, recycle internally reflected photons without sacrificing mechanical compliance [74,85]. These features are particularly beneficial for wearable or implantable PBM systems, where conformability is essential. Recent innovations in metasurfaces offer precise spatial and spectral control of NIR emission. Engineered sub-wavelength resonator arrays enable beam shaping and polarization tuning, while chiral plasmonic ceramics facilitate circularly polarized light emission, aiding selectivity for PBM in neuro stimulation [86,87,88].

Plasmonic heating and spin-antenna concepts have also been reported in related optoelectronic systems, and in the context of NIR LED-stack geometries, such plasmonic nanostructures can both enhance light extraction and influence thermal load within wearable arrays, where the emitter–plasmon distance critically determines the balance between radiative enhancement and heat dissipation [89]. Likewise, spin-antenna effects can be understood in engineered cavity–emitter alignment within layered LED stacks [90], promoting directional emission and spectral selectivity for PBM applications. In parallel, the broader field of NIR optoelectronics contributes additional insights into device performance optimization. Miao et al. [69] demonstrated that microcavity top-emission architectures significantly boost light outcoupling efficiency in perovskite LEDs, reaching an EQE of 20.2%. Their angular emission analysis confirmed the role of optical interference effects in maximizing photon extraction. Similarly, Zhang et al. emphasized the outcoupling bottleneck as a major challenge even when the internal quantum efficiency approaches unity.

Device speed and bandwidth are also essential for advanced PBM systems. Takeda et al. demonstrated photonic crystals that achieve a modulation bandwidth of 17.8 GHz, support 25 GHz direct modulation, and operate with ultralow energy making it exceptionally efficient and fast for on-chip or short-range optical interconnect applications [82]. Such technology can be adapted to NIR LEDs for fast-switching PBM applications, e.g., neurostimulation. We expect that for the future, the convergence of photonic, plasmonic, and optoelectronic engineering will drive the development of high-performance NIR LEDs for PBM. From metasurfaces to microcavity designs, these advancements enable not only enhanced emission but also intelligent and personalized therapeutic capabilities.

## 5. Concluding Remarks and Perspectives

NIR LEDs hold tremendous promise for advancing non-invasive PBM, owing to their ability to deliver therapeutic light to deep tissues with minimal side effects [1,2]. As PBM continues to demonstrate efficacy in treating conditions ranging from chronic wounds to neurodegenerative diseases [4,17], the demand for efficient, safe, and flexible NIR emitters is growing rapidly. In this context, perovskite-based NIR LEDs have emerged as a compelling alternative to traditional inorganic semiconductors and organic emitters. Lead halide perovskites offer narrowband emission and high photoluminescence quantum yields, while tin-based, lanthanide-doped, and vacancy-ordered perovskites extend emission into clinically relevant NIR wavelengths [12,13,58]. However, material instability, particularly under operational stress, remains a persistent barrier. Further research into oxidation-resistant compositions and passivation strategies will be essential for device longevity [54,59]. The integration of photonic and plasmonic engineering represents a synergistic pathway to overcome the intrinsic limitations of NIR materials. Optical cavity designs, dipole alignment, and plasmonic nanostructures have already shown promising enhancements in light outcoupling and emission intensity [14,74,80]. In the future, metasurfaces, anisotropic nanostructures, and thermally adaptive photonic platforms offer exciting frontiers for dynamic spectral control [86,91]. From a translational perspective, biocompatibility and toxicity remain central concerns for clinical deployment. Recent lead-free perovskites are gaining attention as safer alternatives, but their performance in long-term and in vivo settings is not yet fully validated [57,66]. Comprehensive toxicological studies and scalable synthesis methods are critical next steps toward clinical integration. In summary, the convergence of advanced materials science, photonic engineering, and biomedical research is catalyzing the development of next-generation NIR LEDs tailored for PBM. Interdisciplinary collaboration will be pivotal in transforming laboratory-scale innovations into practical, wearable, and patient-friendly therapeutic tools. Future work should prioritize materials stability, device reliability, spectral specificity, and biocompatibility to fully unlock the therapeutic potential of engineered NIR light.

## Figures and Tables

**Table 1:** Representative PBM clinical and preclinical studies for wound healing, neurological, psychiatric, and retinal conditions. An asterisk (*) indicates animal studies outside ophthalmology.

Subjects	Wavelength (nm)	Fluence (J/cm^2^)	Irradiance (mW/cm^2^)	Duty Cycle	Session Time	Ref.
Burn wounds *	785, 830	3	8.5	Not specified	Single exposure	[31]
Dermal wounds	810–830	1–6	5–50	CW/ PW	Multiple sessions	[2]
Full-burn wounds *	904	0.2	0.4	100 Hz PW	7 daily sessions	[32]
Diabetic wounds *	904	18.3	304.8	9500 Hz PW	5 daily sessions	[5]
Acute stroke	808	1.2	10	CW	2 min each side	[28]
Traumatic brain injury	810, 980	14.8–28.3	10–15	10 Hz PW	Multiple sessions	[29]
Depressive disorder	810	60	250	CW	4 min sessions	[30]
Age-related macular degeneration	780	0.3	0.3	292 Hz pulse	40 s 2 weeks	[18]
Amblyopia	780	0.7	0.3	292 Hz pulse	30 s 2 weeks	[22]
Retinis pigmentosa	780	0.4	0.3	292 Hz pulse	40 s 2 weeks	[23]

**Table 2:** Worked example converting radiance and angular profile to skin-plane irradiance and radiant exposure.

Device radiance, *L*	$10\;W\;{\mathrm{sr}}^{-1}\;{\mathrm{cm}}^{-2}$
Angular profile	Lambertian, half-angle $60^{\circ }$
Spot area	1 cm^2^
Calculated irradiance, *E*	31 W/cm^2^ (31,000 mW/cm^2^)
Duty cycle	50% → $E_{\mathrm{avg}}\approx 16,000\;\mathrm{mW}/{\mathrm{cm}}^{2}$
Session time	60 s
Delivered radiant exposure	940 J/cm^2^

**Table 3:** Comparison of different LED material classes for NIR photobiomodulation (700–1000 nm). Parameters: emission peak ($\lambda_{\mathrm{peak}}$), full-width at half maximum (FWHM), external quantum efficiency (EQE), wall-plug efficiency (WPE), radiance/irradiance at treatment plane (Rad./Ir.) in (mW/cm^2^) ^†^, current density at EQE roll-off ($J_{\mathrm{ro}}$), spectral drift (S) in (A/cm^2^) ^‡^, $LT_{50}$ with CW test conditions, surface temperature rise (ΔT), and references (Ref.).

Material Classes	*λ*_peak_ (nm)	FWHM (nm)	EQE (%)	WPE (%)	Rad. /Ir. ^†^	*J*_ro_ ^‡^	S (nm)	*LT*_50_ (h)	Δ*T* (°C)	Ref.
III–V (GaAs)	700–950	20–40	30–72	25–35	50–100	1–5	<2	>10^3^	5–8	[67]
IV (SiC)	900–1000	50–80	1–5	0.5–2	5–20	0.1–0.5	>5	<100	3–5	[38,71]
II–VI (CdSe)	750–875	25–35	10–21	10–15	10–50	0.5–1	2–4	∼500	5–7	[10,41]
Small molecule	700–850	40–60	5–10	3–6	5–15	0.1–0.3	5–10	100–300	2–4	[10,43]
Polymer	700–950	50–80	2–20	1–3	2–10	0.05–0.2	5–15	<100	2–4	[10,49]
Perovskite (pure)	700–986	20–30	10–31	10–12	20–50	0.2–1	1–3	200–500	3–6	[11,12]

## Data Availability

No new data were created or analyzed in this study. Data sharing is not applicable to this article.
